# Genome Sequence of the Oral Probiotic *Streptococcus salivarius* JF

**DOI:** 10.1128/genomeA.00971-16

**Published:** 2016-09-22

**Authors:** Fang Jia

**Affiliations:** Department of Stomatology, Xintai People's Hospital, Xintai, China

## Abstract

*Streptococcus salivarius* is a nonpathogenic Gram-positive bacterium and the predominant colonizer of the oral microbiota. It finds a wide application in the prevention of upper respiratory tract infections, also reducing the frequency of other main pathogens. Here, we present the complete genome sequence of the oral probiotic *S. salivarius* JF.

## GENOME ANNOUNCEMENT

The probiotic strain *Streptococcus salivarius* was originally isolated from the saliva of a healthy child and could produce several bacteriocin-like inhibitory substances, such as the lantibiotics salivaricin A and salivaricin B (1). *S. salivarius* is also the prototype species of the *S. salivarius* group, which includes the important dairy species *S. thermophiles* (2). As *S. salivarius* is generally associated with good oral health, several bacteriocinogenic strains with proven safety records have been developed as oral probiotics (3, 4). *S. salivarius* JF was isolated from a child’s saliva that is able to produce various bacteriocins. Here, we present the complete genome sequence of this strain.

Genomic DNA from *S. salivarius* JF was extracted using the Wizard Genomic DNA purification kit (Promega). The quantity and quality of genomic DNA were evaluated on the Bioanalyzer 2100 (Agilent). A 10 kb insert single-molecule real-time (SMRT)-bell library was constructed and then was sequenced by Pacific Biosciences (PacBio) RS II sequencer (Pacific Biosciences, CA) (5). A total of 98,133 polymerase reads on one SMRT cell for 3-h movie times led to a total of 1,373,047,040 nucleotide bases. After filtering to remove any reads having accuracy values less than 0.8, 1,215,197,525 read bases were obtained. All of the filtered sequences were *de novo* assembled using SMRT analysis software version 2.3.0 (Pacific Biosciences) (6), which resulted in one circularized complete chromosome sequence, with more than 270-fold coverage. The open reading frames (ORFs) were predicted with functional annotation and metabolic analysis performed with the Rapid Annotation using Subsystems Technology (RAST) server (7). The total size of 2,191,044 bps genome is composed of one circular chromosome with a G+C content of 40.2%. The coding regions cover 86.5% of the genome, including 1,944 protein coding genes (CDSs), 23 tRNAs, and 92 rRNAs.

According to the results of RAST, we found 319 subsystems, including 209 (17%) and 15 (10%) genes related to amino acids metabolism and carbohydrate metabolism. In the genome of JF, we identified multiple genes for the bacteriocin production, such as 2 genes encoding BlpU and 5 genes related to the production of colicin V. In the strain we also found two genes encoding dextranase (EC: 3.2.1.11) which hydrolyzes D-glucosidic linkages of the exopolymeric substances of *S. mutans*. This may improve the anti-*S. mutans* inhibitory activity of the bacteriocin. The multiple bacteriocin and dextranase activities of strain JF support its candidature for development as an oral probiotic. The *S. salivarius* JF genome sequence will not only be useful for comparative genomics but is essential for the development of a functional genomics platform facilitating molecular evolution.

### Accession number(s).

This whole-genome shotgun project has been deposited at DDBJ/EMBL/GenBank under accession number CP014144.
